# Outpatient Clinical Trial in Dogs With Leptospirosis Treated With Enrofloxacin Hydrochloride-Dihydrate (ENRO-C)

**DOI:** 10.3389/fvets.2019.00360

**Published:** 2019-10-15

**Authors:** Lilia Gutierrez, Jesús Mendoza, Ana Bertha Rangel, Graciela Tapia, Maria Josefa Bernad, Hector Sumano

**Affiliations:** ^1^Department of Physiology and Pharmacology, Veterinary Medicine Faculty, National Autonomous University of Mexico, Mexico City, Mexico; ^2^Department of Genetics and Biostatistics, Veterinary Medicine Faculty, National Autonomous University of Mexico, Mexico City, Mexico; ^3^Department of Pharmacy, Chemistry Faculty, National Autonomous University of Mexico, Mexico City, Mexico

**Keywords:** treatment, leptospirosis, dogs, enrofloxacin hydrochloride-dihydrate, enro-C

## Abstract

Pharmacokinetics of enrofloxacin HCl-2H_2_O (enro-C) in dogs and Monte-Carlo simulations against *Leptospira* spp. prompted a clinical study to treat the clinically apparent phase of this disease. Leptospirosis was diagnosed by real-time PCR from blood, micro-agglutination titers (MAT), clinical signs and blood parameters of the liver and kidney. In order to determine the clinical ability of the participants to diagnose leptospirosis on the first exam and establish an early treatment to avoid excessive organ damage, patients were clinically classified as: high-risk or medium-risk. Forty-five dogs were included in this trial (from 2017 to early 2019). The treatment consisted of IM injections of a 5% aqueous enro-C suspension (10 mg/kg/day) for 10 days, and subsequently enro-C was administered orally for another 7 days in gelatin capsules. Thirty-four high-risk and 11 medium-risk dogs were treated, including 6 puppies (4 high-risk with ages between 6 to 10 months and 2 medium-risk dogs with an average age of 6 and 7 months). Other ages ranged from 1 to 5 years. Fifteen cases had a history of having received prior treatment with other antibiotics, including all puppies. The clinical diagnostic error was 13.5% (7/52 cases), and only one of the misdiagnosed dogs had been classified as a high-risk patient. Three to 5 days after finishing treatment with enro-C, 82.2% of the dogs were negative to real-time PCR from urine samples and 100% negativity was observed on day 30 after treatment, when antibody titrations dropped to 1:100–1:200. Based on the absence of clinical signs, real-time PCR, and MAT titers, all treated dogs were considered as successful treatments. Within 6–24 months of clinical follow-up, no relapses were recorded. Adverse effects were inconsequential. This study represents the first report of a successful treatment of canine leptospirosis using a fluoroquinolone, and due to its efficacy, it is suggested that enro-C be considered as a viable option for the treatment of this disease.

## Introduction

Leptospirosis has been considered as the most widely distributed zoonosis (1), with the highest density in tropical and subtropical areas (1, 2). It is a common pathology in canine medicine, particularly in tropical and subtropical areas of the world. It has been stated that, despite vaccination, a greater number of cases have been observed in the last decades with various clinical presentations (3). Although immunization limits the spread of this disease, the efficacy to contain it has had poor performance due to defective compliance and the questionable adequacy of vaccine practices in the field (4). There are more than 250 serovars of *Leptospira* spp., and many are pathogenic for dogs, especially the *Leptospira interrogans* serovars Icterohaemorrhagiae, Canicola, Pomona, Australis, Sejroe, Atumnalis, Djasiman, and Ballum; and also the *L. kirshneri* serovar Grippotyphosa and *L. noguchii* (5). Infected animals become bacteremic for different periods of time and *Leptospira* spp. multiplies in the kidney, liver, spleen, central nervous system, eye tissue, and genital tract (6). Host-reservoirs can show a subclinical form of the disease and can eliminate microorganisms for months or years before they eventually relapse.

An ideal treatment for leptospirosis is still lacking (7). If an early diagnosis is achieved in dogs, the administration of large daily doses of procaine benzylpenicillin G, over weeks, may achieve a bacteriological cure and limit organ damage (8). If liver damage occurs, ampicillin or amoxicillin should be added to the treatment (9, 10). In most cases, doxycycline has been considered the gold-standard treatment for canine leptospirosis. It is recommended for 14–21 days at a dose of 5–10 mg/kg/day orally, administered with food (5). This treatment is often interrupted by the adverse effects of doxycycline in the gastrointestinal tract, such as severe esophageal vomiting and irritation (11). If this last scheme fails, there are not many options available (12). In addition, bacteriological cure is not always achieved and animals can become chronic carriers of *Leptospira* spp. (8, 9).

Fluoroquinolones are not commonly used to treat canine leptospirosis. However, in a hamster-model study, high doses of ciprofloxacin or levofloxacin (50 mg/kg/day) and standard doses of gatifloxacin (5–25 mg/kg/day), produced a statistically significant survival advantage compared to no treatment and demonstrated a survival similar to the one observed with doxycycline therapy (7). Nevertheless, fatalities due to diarrhea were reported as a serious drawback in this study. Orbifloxacin was ineffective in a dog with leptospirosis (8). In its technical manuals, the pioneer brand of enrofloxacin, does not mention its possible use to treat canine leptospirosis^1^, nor was this fluoroquinolone considered as an option in the consensus statement published by experts (5). In contrast, favorable pharmacokinetics (PK) of a recrystallized form of enrofloxacin such as HCl-2H_2_O (enro-C) in dogs, its pharmacokinetic/pharmacodynamic ratios (PK/PD) and Monte-Carlo simulations, indicate that its IM administration may result in concentrations that may be appropriate for the treatment of leptospirosis (13). These data were the motivation to carry out this outpatient clinical trial with dogs affected by leptospirosis and treated with enro-C.

## Materials and Methods

### Animals

All study procedures and animal care activities were conducted in accordance with the Institutional Committee for Research, Care and Use of Experimental Animals of the National Autonomous University of Mexico (UNAM), in accordance with Official Mexican Regulation NOM-062- ZOO-1999 (14). In this study, only dogs owned by the client (*n* = 45) were eligible for inclusion. The patients were recruited from three hospitals in Mexico City and those referred to the Pharmacology Department of the Veterinary Medicine Faculty from the National Autonomous University of Mexico, also from Mexico City. Written informed consent forms were provided to all dog owners, including communications relevant to the enrollment of an animal patient in this clinical study, full disclosure of the study purpose, associated risks and benefits, potential adverse drug reactions, study design and necessary medical interventions. Dog owners had adequate time to consider participation without real or perceived coercion; then, they decided whether to sign or not. Only animals with signed forms were included. It was not possible to set a positive control group treated with doxycycline since most owners rejected the idea of possible adverse gastrointestinal effects, because the dogs already suffered from hyporexia or anorexia, and oral administration of doxycycline was difficult at best. Nor was it possible to establish an untreated control group, based on ethical considerations (15, 16). Therefore, a longitudinal open labeled clinical trial was carried out.

Upon arrival, a complete owner-based anamnesis was performed. The dogs were examined clinically and in order to determine the clinical capacity of the participants to diagnose canine leptospirosis without laboratory data, the patients were clinically classified according to the common clinical signs of leptospirosis, such as fever, joint or muscle pain, decreased appetite, weakness, vomiting and diarrhea, nasal and ocular secretion, frequent urination, yellowing of the gums, and conjunctiva. Then, a scoring system was designed to diagnose canine leptospirosis as high-risk (range: 15–19 points), medium-risk (range: 6–14 points), and low-risk (range: 0–5 points) (see Table 1). Uveitis was not considered since none of the dogs presented this sign on the ophthalmoscopic examination.

**Table 1:** Scoring system for assigning a risk value composite endpoint (17) to a dog suffering from leptospirosis.

**Clinical case diagnosis**	**Clinical signs**				**Micro-agglutination test titers**		**Real-time PCR**
	**Renal damage^†^**	**Degree of liver damage^‡^**	**Degree of musculo-skeletal disease^§^**	**Degree of urinary changes^¶^**	**With a vaccination history**	**Without a vaccination history**	
High risk^a^	3	3	3	3	3 (≥ 400)	3 (≥ 200)	4 (positive)
Medium risk^b^	2	2	2	2	2 (200)	2 (100)	4 (positive)
Low risk^c^	1	1	1	1	1 (negative)	1 negative	0 (negative)

† Severity of renal damage was accompanied by various degrees of lethargy, anorexia, vomiting, abdominal pain, and history of polyuria or oliguria, as well as serum creatinine and blood urea nitrogen profiles. Low-risk was associated with minimum changes in all variables and normal blood parameters (1); medium-risk presented some clinical signs and borderline or slightly higher levels of creatinine, whereas the high-risk presented clearly most or all clinical signs and urea and creatinine levels > 1.4 mg/dl (reference range 0–1.6 mg/dl).

‡ Dogs may be icteric; hepatic blood profile reveals elevated bilirubin (reference range: <0.4 mg/dl) and ALP (alkaline phosphatase) (reference range: 15–127) and sometimes ALT (alanine aminotransferase) (reference range: 10–130) and/or AST (aspartate transaminase) (reference range: 15–43). Grading liver damage followed the same criteria as for kidney damage.

§ Muscle pain, stiffness, weakness, trembling, or reluctance to move.

¶ Hypo or hypersthenuria, proteinuria, glucosuria, cylindruria, hematuria, pyuria, revealed by urinalysis.

a,b,c *Sum of scoring ranges in (15–19), (6–14), and (0–5) for high, medium, and low risk of having leptospirosis, respectively*.

### Microbiological Studies

Two blood samples of 2–3 ml were obtained and sent to the Department of Microbiology (UNAM) in order to determine micro-agglutination titers (MAT) (18) and real-time PCR. The main agglutinating serovars of anti-Leptospira antibodies to identify were: Autumnalis, Bataviae, Bratislava, Canicola, Celledoni, Grippotyphosa, Hardjo, Icterohaemorrhagiae, Pomona, Pyrogenes, Tarassovi, and Wolffi. In addition, blood samples 3–5 days after treatment and 30 days later were sent for MAT, and urine samples were processed for real-time PCR on the same days. Renal and hepatic blood profiles and complete hematological tests were also carried out on the same days. A urinalysis was also performed on the same day; the owners collected the sample in a clean and sterile container, waiting for the dog to urinate. Isolation of *Leptospira* spp. microorganisms was not attempted.

### Inclusion Criteria

Inclusion criteria corresponded to dogs with signs of leptospirosis, high MAT, ideally >800, but often only >400 (5), and positive to real-time PCR in blood samples (17). Exclusion criteria were based on dogs with low MAT titers (<200) and negative real-time PCR results. Due to the uncontrolled, ambulatory and experimental nature of this trial, dogs with severe renal and/or hepatic impairment requiring hospitalization were not included.

For the PCR test, DNA extraction was performed using QIAamp DNA Mini kits, following the manufacturer's instructions (Qiagen México S. de R.L. de C.V., Mexico City) with a final volume of 200 μl. Primers were designed as reported by Stoddard (19): LipL32-45F (5′-AAG CAT TAC CGC TTG TGG TG-3′) and LipL32-286R (5′-GAA CTC CCA TTT CAG CGA tt-3′). Real-time PCR was conducted using TaqMan PCR Master Mix in a volume of 20 μl containing 400 nM of forward primer, 400 nM of reverse primer, 12.5 μl of Master Mix, and 5 μl of DNA clinical extract. The amplification protocol consisted of 5 min at 94°C followed by 40 cycles (30 s at 94 °C, 30 s at 68°C, 30 s at 72°C). After the reaction, the samples were cooled at 40°C for 120 s.

Because the MAT and PCR results were made available to us in ~5 to 10 days, all dogs were initially treated with enro-C, but in this study only positive real-time PCR dogs were included; however, the clinical scores (Table 1) allowed recruitment of patients with MAT values <800. Initial clinical scores were also considered to assess clinical improvement or lack thereof. If no improvement had been detected, treatment failure would have been declared, registered and the dog would have changed to a different antibacterial scheme. This eventuality did not occur. On day 30, after completing the dosing scheme for enro-C, failure of the bacteriological cure was assumed if the urine real-time PCR was positive.

### Antibacterial Treatment

Batches of recrystallized enrofloxacin were prepared as indicated in Patent 472715 (*Mexico/Instituto Mexicano de Protección Industrial*: *IMPI MX*/a/2013/014605 and PCT/Mx/2014/00192, Mexico City). This process produces enrofloxacin hydrochloride-dihydrate, identified as enro-C. Enrofloxacin with a purity of 99.97% was purchased from Globe Chemicals (Mexico). For IM injection, a 5% suspension was freshly prepared with sterile water, with a measured pH of 6.5. The selected injection sites were in the semitendinosus or semimembranosus muscles, injecting volumes of 1 to 3 ml per injection site, at a dose of 10 mg/kg/day for 10 days. After completing the parenteral treatment, an oral follow-up was established for an additional 7 days using custom-made gelatin capsules, which were also administered at a dose of 10 mg/kg.

Because this study was an outpatient clinical trial, no dogs were hospitalized, so owners were instructed to disinfect and clean the dog's habitat; they were also given an information booklet to warn them about the potential dangers of this zoonosis and how to prevent it. In addition to the clinician, responsible for daily injections of enro-C, owners were instructed to monitor any adverse reactions in their dogs, including allergic reactions, ataxia, depression, seizures, pruritus, mood swings, appetite changes, and of course, pain at the injection site. This information would have been recorded as an adverse event.

### Statistical Analysis

Clinical signs before and after treatment with enro-C were compared using a Wilcoxon matched-pair rank test with Z approximation (20). Antibody averages from both strata (high-risk and medium-risk dogs) were analyzed by re-sampling a stratified weighted bootstrap from the results of the original paired *t*-test (21). A repeated sample ANOVA analysis was performed for the biochemical analytes; while for the urinalysis, a Z test for two proportions was carried out. The IBM SPSS package was used. A statistical significance level of 0.05 was accepted.

## Results

**Figure 1:**
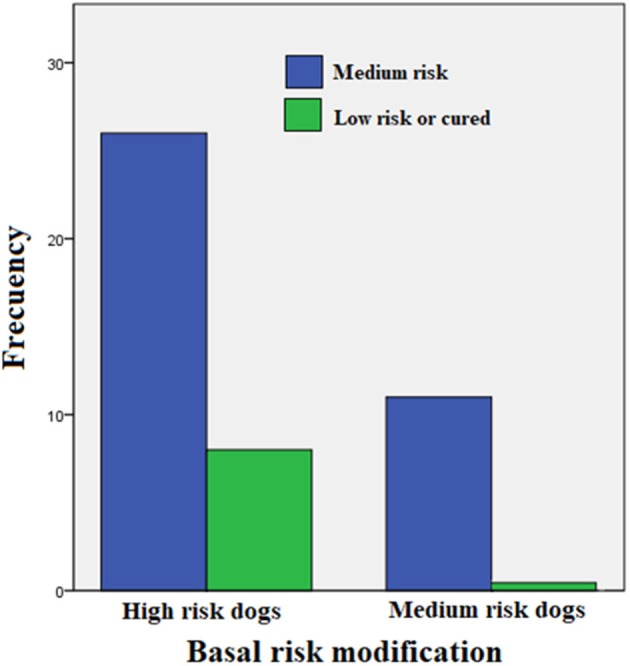
Change in the risk classification of dogs suffering leptospirosis and their change after treatment (Wilcoxon matched-pair rank test: Zc = −4.686; *P* = 0.0001).

Table 2 shows a summary of the cases treated, grouping dogs according to their age and classification strata. This trial included only 45 PCR-positive clinical cases of the 52 initially recruited (23 Mongrel dogs, 2 Belgian Tervuren, 3 Boxer, 1 Cocker Spaniel, 1 Collie, 1 Dalmatian, 4 Doberman, 3 German Shepard, 1 Giant Schnauzer, 2 Labrador Retriever, 2 Scotish Terrier; 1 Weimaraner and 1 Xoloitzcuintli). Six puppies between 6 and 10 months (4 mongrel dogs, 1 Cocker Spaniel, and 1 German Shepard) were treated with enro-C and all were classified as high-risk of suffering from leptospirosis. Despite the known contraindications of the use of enrofloxacin in growing dogs, it was decided to treat them with enro-C, based on their history of failure to complete a previous treatment with doxycycline. Twenty-eight additional dogs were also stratified as high-risk cases. In this group, 8 additional dogs had a history of failed treatment with doxycycline alone or with beta-lactamic antibiotics. Eleven dogs were considered medium-risk. One of them was previously and unsuccessfully treated with doxycycline. In the end, based on real-time-PCR data, an overall clinical diagnostic error of 7 out of 52 cases (13.5%) was considered, and only one misdiagnosed dog had been classified as a high-risk patient. Figure 1 shows the change in the risk ranking of dogs suffering leptospirosis and their change after treatment by means of a Wilcoxon matched-pair rank test (Zc = −4.686; *P* = 0.0001).

**Table 2:** Summarized description of the 45 dogs treated with enrofloxacin hydrochloride-dihydrate (enro-C) against leptospirosis.

**No. of** **dogs/age** **(years)**	**Initial certainty in diagnosis****^†^**	**immunized/not immunized**	**Mean difference** **±** **SE** **antibody titers (95%CI)**^¶^		**Real-time PCR**^**§**^ **Negative/Positive** **(per cent)**		**No. of dogs/No. of weeks treated with enro-C**		**Previous treatment**
			**Before Tx/** **3–5 days after Tx**	**Before Tx/** **30 days after Tx**	**3–5 days** **after Tx**	**30 days** **after Tx**	**IM**	**Oral**	**Doxy and/or β-lactam**
6/0.5–0.8; 5/1.0; 3/1.2; 2/1.4; 3/1.5; 8/2.0; 4/2.2; 7/2.5; 4/3.0; 2/3.5; 1/4.5	l34/HR; 11/MR	29/16	607 ± 69 (467,747)	702 ± 71 (560,845)	37/45 (82.2%)	45/45 (100%)	36/2 9/3	45/2	15/7

All dogs were real-time PCR positive at the beginning of the trial. In all cases, treatment^‡^ was considered successful.

† According to Table 1.

HR and MP = highly-risk or medium-risk affected by leptospirosis.

^‡^Tx = treatment with enro-C 10 mg/kg/day; first 2 or 3 weeks was applied intramuscularly and 2 more weeks orally.

§ All dogs entering this trial were real-time PCR positive.

¶ *Mean differences and 95% confidence intervals (CI), bootstrap results are based on 1000 stratified bootstrap samples*.

**Figure 2:**
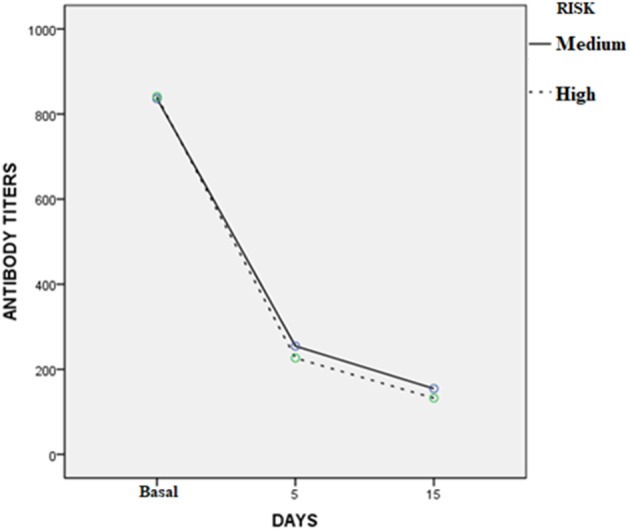
Average change in antibody titration on sampling days in the high and medium risk groups (ANOVA quadratic repeated samples: *F* = 34.1; *P* = 0.0001).

Three to 5 days after the end of treatment, urine samples from 37 of 45 dogs were negative to real-time PCR (82.2%); but 30 days later, all cases treated were PCR negative. For MAT, confidence intervals (95%) of the differences between the means before and 3–5 days after treatment varied from 467 to 747; while for the 30 days after treatment with enro-C, these values varied from 560 to 845. Table 3 shows the mean ± SE of the initial antibody titers and the resulting means 3–5 days and 30 days after the end of treatment. Considering that in 8 cases, 2 or more *Leptospira* serovars were reactive, the total reaction was as follows: 15 Icterohaemorrhagiae; 13 Canicola; 10 Autumnalis; 8 Bratislava; 6 Pomona; 4 Pyrogenes; 1 Hardjo and 1 Wolffi. Based on their clinical condition, real-time PCR results and serological conversion, all treated dogs were considered as treatment successes and their clinical follow-up through monthly interviews with dog owners and physical exams, which lasted from 6 to 24 months, confirmed the lack of relapses. Average change in antibody titration on sampling days in both high-risk and medium-risk groups as shown in the ANOVA quadratic repeated samples is presented in Figure 2 (*F* = 34.1; *P* = 0.0001).

**Table 3:** Means and standard errors of antibody titers against *Leptospira* spp. before and 3–5 or 30 days after ending the treatment with enro-C.

**Initial certainty in diagnosis**	**Mean** **±** **SE antibody titers**		
	**Before Tx**	**3-5 days after ending Tx**	**30 days after ending Tx**
34/HR	841 ± 81^a^	226 ± 19^b^	132 ± 8^c^
11/MR	836 ± 157^a^	254 ± 37^b^	154 ± 16^c^

HR and MR = diagnosis of high-risk or medium-risk of having leptospirosis.

SE = standard error.

a,b,c *Significant differences between rows P = 0.001, bootstrap results are based on 1,000 stratified bootstrap samples of paired t-tests*.

Laboratory data are summarized in Table 4 as overall means and standard deviations. No statistically significant differences were observed between the risk groups. No dermatological adverse events were detected at the injection sites. The six puppies included in this trial did not show apparent adverse effects on their joints. Mild and transient gastrointestinal disorders were reported, with slightly loose stools in three of the 45 cases treated.

**Table 4:** Summary of blood-liver enzymes and renal profiles of dogs affected with leptospirosis and included in the treatment trial with enrofloxacin hydrochloride-dihydrate (enro-C).

**Variable**	**Mean** **±** **SD**			**Reference range**
	**Before Tx**	**3–5 after Tx**	**30–35 days after Tx**	
Total bilirubin (mg/dl)	1.68 ± 0.75	1.00 ± 0.58	0.65 ± 0.35	<0.4
ALP(U/l)	175.77 ± 82.29	149 ± 72.83	92.4 ± 37.47	15–127
ALT (U/l)	146.02 ± 45.67^a^	132.37 ± 52.76^a^	55.4 ± 25.54^b^	19–70
AST (U/l)	134.48 ± 45.90^a^	101.93 ± 41.19^a^	40.57 ± 16.94^b^	15–43
Creatinine (mg/dl)	3.92 ± 0.86	2.23 ± 0.80	1.10 ± 0.40	0.6–1.4
BUN (mg/dl)	89.33 ± 21.21^a^	89.33 ± 21.21^a^	44.33 ± 13.44^b^	10–31
**Percentage of patients considered positive**				
Urine protein	68.88% (31/45)^a^	13.33% (6/45)^b^	4.44% (2/45)^c^	−
Urine glucose	22.22% (10/45)^a^	4.44% 2(45)^b^	0% (0/45)	−
Cylindruria	26.66% (12/45)^a^	6.66% (3/45)^b^	0% (0/45)	−
Pyuria	77.77% (35/45)	0% (0/45)	0% (0/45)	−
Hematuria				

a,b,c *Differences in (P < 0.01) for Z tests for two proportions (0% results were not analyzed because of the obvious differences), ALP, alkaline phosphatase; ALT, Alanine transaminase; ST, Aspartate transaminase; BUN, blood urea nitrogen*.

## Discussion

Because this study was designed for outpatient treatment of leptospirosis, cases with anuria, severe oliguria, liver failure, or those requiring therapy with fluids and electrolytes or other supportive measures were excluded. In these patients, the numerous pharmacological interventions that these dogs may have needed would not have allowed clear conclusions regarding the efficacy of enro-C. However, a study with more critical patients is required.

The close follow-up by the investigators in all cases ensured compliance with the enro-C treatment and allowed the thorough inspection of each individual. To minimize the spread of leptospirosis infections in humans, each owner was instructed on the disinfection of the pet's environment and the use of gloves to handle the urine and feces of the animal. However, it is important to note that the normal daily activities of owners and pets seem to show only a moderate risk of leptospirosis infection (22).

Given the severity of leptospirosis, it is important to note that the establishment of an untreated control group was not considered ethical in this study (15). In addition, it was not possible to gather a sufficient number of dogs in a positive control group treated with doxycycline, the alleged gold-standard treatment of canine leptospirosis, so far accepted (5). The owners understood the purpose, risks and potential benefits of the study (23), but potentially adverse reactions to doxycycline (12) and, in some cases, the lack of previous results alone or combined with a β-lactamic derivative (24), caused the rejection of participating and allowing their pets to be part of this control group. However, once the first cases of leptospirosis were resolved with enro-C, it was much easier to complete the study with this medicine since the owners intentionally sought this treatment and canceled any other. Therefore, instead of comparing treatments, the baseline data measurements of all the tested parameters were compared with the corresponding data obtained after the treatment. The use of a single-composite endpoint in this study was considered ideal, since the treatment showed benefits in all components. The increase in statistical potency helped confirm (or deny) an overall benefit when treating dogs with enro-C, minimizing the influence that a particular endpoint would have had on the significance of these results, simply by chance (17). Although, further statistical analysis should be carried out in an additional study, since the therapeutic effects of enro-C may be influenced by age and individual factors in dogs.

It is important to emphasize that the collection of 45 cases of leptospirosis in this study may be related to the lack of timely vaccination of dogs (usually annual), or the insufficiency of serovars contained in commercially available vaccines in Mexico (usually Canicola, Icterohaemorrhagiae, Pomona, and Grippotyphosa). Some studies suggest that protection does not last long (25); particularly in humid tropical and subtropical geographical areas (5). Furthermore, the immunity produced by vaccination is known to be limited to the specific serovars included. Vaccines formulated with international reference strains, may not generate adequate protection against native strains (26). After experimental immunization in dogs with commercial vaccines from international laboratories, the results indicate that they provide protection against clinical signs, but do not necessarily prevent leptospiremia and the development of renal carriers (27). Therefore, the suitability of the antigenic determinants used in the vaccines available in Mexico can be reviewed.

Martin et al. (28) and Miotto et al. (29) found that antibody titers against *Leptospira* spp. were often negative during the clinical phase of the disease and, at best, the titers ranged between 100 and 200 six months after vaccination. Therefore, unlike some cases of leptospirosis in humans (30), antimicrobial therapy in veterinary medicine is indicated in all suspected cases of leptospirosis. This therapy should be started as soon as a presumptive diagnosis of leptospirosis is established and should be performed as a complete course, regardless of the initial serological results. In this context, it is not surprising to have obtained low antibody titers in the basal samples of this trial, since it has been claimed that a single negative or low MAT titer cannot be used to rule out leptospirosis in the early stages of the disease. Seroconversion usually, but not always, occurs within 8 days (31). Hence, if leptospirosis is suspected and the initial MAT is not confirmatory, a convalescent MAT should be attempted, usually 2, maximum 4 weeks later (32). This serological behavior is in agreement with our MAT results, whose average before treatment was below the usual accepted cut-off titer of 800. Therefore, the patients in our study were diagnosed as positive for leptospirosis, through the combination of hematological and clinical signs, real-time PCR analysis and the existence of unconventional serological profiles through MAT. These last two tests were available ~5 to 10 days after the initial clinical examination. For this reason, all dogs that went to the veterinarian's office and were clinically diagnosed as suffering from leptospirosis were treated with enro-C. However, it is important to emphasize that only those patients that were subsequently confirmed positive to real-time PCR were included as data in this trial. The handling of patients in this way meets the available criteria that recommend early treatment to achieve the best clinical results (5), and because no standard method is currently accepted as reliable for the on-site detection or clinical diagnosis of *Leptospira* spp. (33–35). In this trial no cases of uveitis were detected. This coincides with the literature, since only few reports describe cases of uveitis due to canine leptospirosis (36). In contrast, mild conjunctivitis and ocular-nasal discharge were more common, but these data were not quantified in this study, since the differential diagnosis can be excessively complicated and out of place with respect to this trial (8, 36). In any case, the accuracy of the overall clinical diagnosis was 86.5% and misdiagnoses were all but one of the medium-risk dogs.

Currently, there are few experimental studies that allow the selection of antibiotic protocols for the treatment of leptospirosis in dogs. In general, orally administered doxycycline is the drug of choice (37) and occasionally, beta-lactamic drugs, which can be administered alone, sequentially or concomitantly with doxycycline (10, 38). However, the administration of doxycycline has been associated with severe irritation of the gastrointestinal tract (GIT) and this can cause early withdrawal of treatment and, consequently, failure to achieve clinical and/or bacteriological cure (39). Treatment with beta-lactamic drugs has been considered much less effective for treating leptospirosis in humans (40) and dogs (5). To date, there is no parenteral formulation of doxycycline worldwide, and oral administration of doxycycline is not recommended in sick dogs that refuse food, since GIT irritation is often observed after a few doses, and the recommended scheme indicates 21 days of treatment (9, 30, 37). Therefore, it can be stated that there are few antibacterial options available to treat this disease.

Fluoroquinolone drugs show a concentration-dependent activity and the C_MAX_/MIC ≥ 10 ratio is critical to optimize its effectiveness (41). The apparently favorable pharmacokinetics of enro-C in dogs (13), and its almost 100% efficacy in bacteriological cure in an experimental model of hamster leptospirosis (42), prompted the use of enro-C in this trial. The chosen dose of enro-C at 10 mg/kg/day IM, followed by oral administration, was based on achieving the aforementioned ratio through previous Monte-Carlo simulations for dogs (13). Such simulations applied to pharmacokinetics reduce uncertainty, when using a given dose in a clinical scenario and allow a therapeutic prognosis if the referred C_MAX_/MIC ≥ 10 target is reached. Therefore, smaller doses were not attempted. In addition, it was thought that a relatively long treatment scheme was necessary given the zoonotic nature of the disease and to avoid the possibility of ending up with a patient suffering from chronic leptospirosis (29). Also, it is important to mention that the referred Monte-Carlo simulations were derived from PK data generated in healthy dogs. It would be interesting to carry out Monte-Carlo simulations with PK data from critical patients, such as those infected with *Leptospira* spp., since the disease status can influence the PK of therapeutic drugs. In addition, a relatively high dose of enro-C was selected from the Monte-Carlo simulations. This complies with most authors who suggest the highest possible dose administration when selecting a fluoroquinolone. In this way, the so-called mutant preventive concentrations are more likely to be obtained and, in turn, this will reduce the generation of strains resistant to these antimicrobial drugs (41, 43, 44).

The treatment of puppies with enrofloxacin has been linked to the development of tendinopathies, including spontaneous tendon rupture. Young dogs are also prone to cartilage damage; particularly, when high tissue concentrations are reached (10–200 μg/ml) (45). Therefore, the use of enrofloxacin is contraindicated in dogs during their rapid growth phase (between 2 and 8 months of age). Despite the above and due to the severity of this disease, six puppies from 6 to 10 months of age, unsuccessfully treated previously with doxycycline, were included in this study. No gait abnormalities were observed in any of them, after 10 days of enro-C IM and 7 days of enro-C administered orally. However, the lack of specific studies to assess the toxicity of enro-C in skeletal or tendinous structures imposes the need for additional studies to characterize the possible damage induced to these structures by this drug and to ponder the risk/benefit ratio of using enro-C for the treatment of leptospirosis in puppies. No other important side effects were observed and there was an absence of observable adverse reactions at the injection sites. This was also reported for enro-C in hamsters. Measured parameters of renal and hepatic blood, as well as urinalysis revealed no apparent side effects. These parameters showed an almost total recovery, except in two cases, in which proteinuria was still present 30 days after the end of treatment. This last finding may have been induced by glomerular damage caused by leptospirosis. Therefore, according to the results shown in Table 4, it seems that most of the test variables were still outside the range of 30–35 days after treatment. Further studies are needed to evaluate these variables beyond the established time period, to help characterize organ damage by leptospirosis and the role of the administration of enro-C.

From the authors' perspective, the challenge trials of leptospirosis induced in healthy dogs are ethically incorrect and require clinical trials with real cases (5, 46). In addition, setting a group treated with doxycycline was considered unfair to dogs in that group, since GIT side effects are very common. Paulus et al. (15), suggested that under some conditions single group studies provide useful information on the comparative effectiveness of the interventions, one of them an implicit comparison where either: the expected course of the disease is known with almost certainty and the effect observed in the study group is evident or the magnitude of the changes observed after treatment in the study group is indisputable. Both circumstances apply to the present study in which all patients were carefully examined to obtain a reasonable source of evidence. However, more studies are required to extrapolate these findings to a diverse population of dogs suffering from leptospirosis in different clinical settings. In addition, an exhaustive search in the available literature shows the lack of formal efficacy studies to evaluate the treatments of canine leptospirosis. Therefore, cure rates achieved with beta-lactamic drugs or doxycycline are not available.

The only exception is a study that reports that even after the administration of doxycycline or amoxicillin, 40% of the patients had to be sacrificed for progressive clinical signs and 33% of the remaining dogs remained positive for leptospirosis, as diagnosed by PCR (47). Another report showed that conventional treatments in a dog affected by leptospirosis, both with amoxicillin and doxycycline, failed to cure it neither bacteriologically nor clinically (39). Considering the above, the conclusion reached in this study is that the daily IM injection of enro-C (10 mg/kg/day) for 10 days, followed by 7 days of oral administration of the drug in gelatin capsules, is highly effective in treating canine leptospirosis. One hundred percent negativity to real-time PCR from urine samples, 30 days after the end of treatment with enro-C, and a clinical follow-up of 6 to 24 months without complications, support the proposal that bacteriological cure was achieved in all cases. Multicenter studies may reveal the efficacy of enro-C in different clinical scenarios, that is, in cases of canine leptospirosis, unresponsive to other antibacterial drugs.

## Data Availability Statement

All datasets generated for this study are included in the manuscript/supplementary files.

## Ethics Statement

The animal study was reviewed and approved by Committee of Research, Care and Use of Experimental Animals of the National Autonomous University of Mexico (UNAM), according to the Mexican Official Regulation NOM-062-ZOO-1999 (14). Written informed consent was obtained from the owners for the participation of their animals in this study.

## Author Contributions

HS, LG, and JM conceived and designed the study. These authors together with AR carried out the clinical monitoring of patients and their follow ups. MB manufactured enro-C according to GLP standards and GT carried out the statistical analysis. All authors have read and accepted the manuscript as it is presented to the journal.

### Conflict of Interest

The National Autonomous University of Mexico (UNAM), owner of the patent, is open to license the enro-C. The authors declare that the research was conducted in the absence of any commercial or financial relationships that could be construed as a potential conflict of interest.

## Footnotes

^1^Bayer Healthcare LLC. https://www.drugs.com/vet/baytril-enrofloxacin-antibacterial-injectable-solution-2-27-for-dogs.html
